# Y-box protein-associated acidic protein (YBAP1/C1QBP) affects the localization and cytoplasmic functions of YB-1

**DOI:** 10.1038/s41598-018-24401-3

**Published:** 2018-04-18

**Authors:** Ken Matsumoto, Shingo Kose, Iku Kuwahara, Mami Yoshimura, Naoko Imamoto, Minoru Yoshida

**Affiliations:** 1 Chemical Genomics Research Group, RIKEN Center for Sustainable Resource Science, RIKEN, Wako, Saitama, Japan; 2 PRESTO, Japan Science and Technology Agency, Kawaguchi, Saitama, Japan; 3 Molecular Entomology Laboratory, RIKEN, Wako, Saitama, Japan; 4 Cellular Dynamics Laboratory, RIKEN Cluster for Pioneering Research (CPR), RIKEN, Wako, Saitama, Japan

## Abstract

The Y-box proteins are multifunctional nucleic acid-binding proteins involved in various aspects of gene regulation. The founding member of the Y-box protein family, YB-1, functions as a transcription factor as well as a principal component of messenger ribonucleoprotein particles (mRNPs) in somatic cells. The nuclear level of YB-1 is well correlated with poor prognosis in many human cancers. Previously, we showed that a Y-box protein–associated acidic protein, YBAP1, which is identical to complement component 1, q subcomponent-binding protein (C1QBP, also called gC1qR, hyaluronan-binding protein 1 [HABP1] or ASF/SF2-associated protein p32), relieves translational repression by YB-1. Here we show that the nuclear localization of YB-1 harboring a point mutation in the cold shock domain was inhibited when co-expressed with YBAP1, whereas cytoplasmic accumulation of the wild-type YB-1 was not affected. We showed that YBAP1 inhibited the interaction between YB-1 and transportin 1. In the cytoplasm, YBAP1 affected the accumulation of YB-1 to processing bodies (P-bodies) and partially abrogated the mRNA stabilization by YB-1. Our results, indicating that YBAP1/C1QBP regulates the nucleo-cytoplasmic distribution of YB-1 and its cytoplasmic functions, are consistent with a model that YBAP1/C1QBP acts as an mRNP remodeling factor.

## Introduction

Regulation of gene expression in eukaryotes depends on the functions of many proteins that bind DNA and RNA^1^. The activities of these proteins are tightly regulated by multiple mechanisms, including association with ligands or other proteins, post-translational modification, and nucleocytoplasmic transport. Nuclear transport of proteins requires members of the importin superfamily, which act as receptors to transport cargo proteins through nuclear pores (reviewed by^2–4^). These receptors or an adaptor protein, importin α in the case of the receptor importin β, recognize a nuclear localization signal (NLS), a specific stretch of amino acids present in cargo proteins. The receptor–cargo complex interacts with the nuclear pore complex and translocates through the nuclear pore. The nuclear transport process can be recapitulated in a semi-intact cell system in permeabilized cells, using either cytosolic extracts or purified components^5^.

YB-1 is a multifunctional DNA/RNA-binding protein with nuclear and cytoplasmic functions (reviewed by^6–9^). YB-1 was originally identified as a transcription factor that binds to the Y-box sequences in gene promoters, and also functions in DNA repair, alternative splicing in the nucleus through either direct binding to DNA or RNA, or regulation of other trans-acting factors. In the cytoplasm of higher eukaryotes, it functions as a major packaging protein for mRNAs. YB-1 mainly associates with the body of mRNAs, whereas poly(A) binding protein (PABP), another major component of mRNPs, mostly binds to 3′ poly (A) tails of mRNAs^10,11^. YB-1 can repress or activate the translational activity of bound mRNAs dependent on the ratio of YB-1 to mRNA. Stabilization of mRNA is likely to be exerted by the mRNA packaging role of YB-1, thereby making mRNAs inaccessible to translation initiation factors or exoribonucleases^8,12–14^. YB-1 accumulates in the centrosomes in a phosphorylation-dependent manner during G2/M phase, and is required for the centrosome maturation^15,16^. In addition, YB-1 is secreted from cells and thereby acts as a ligand for Notch-3 receptors^17^.

YB-1 is a member of the vertebrate Y-box protein family, which consists of three proteins, YB-1, Contrin, and dbpA^18^. The Y-box proteins consist of three domains: the N-terminal domain, the cold shock domain (CSD), and the C-terminal tail domain^18^. The CSD (~80 amino acids [aa] in length) is highly conserved in the Y-box protein family members, consisting of a five-stranded β barrel that includes the RNP-1 and RNP2 motifs essential for RNA binding. The C-terminal tail domain (~200 aa) consists of four alternating regions of predominantly basic and acidic amino acid clusters (referred to as alternating A/B regions); the acidic regions are also rich in aromatic amino acids. The C-terminal tail domain of Y-box proteins mediates not only their RNA-binding activities, but also their protein–protein interactions. In various types of cultured cells, YB-1 is mostly detected in the cytoplasm, where it is accumulated to processing bodies (P-bodies) under normal conditions and stress granules when cells are exposed to stresses such as heat or oxidative stresses^19^^,^^20^. YB-1 translocates to the nucleus in response to various stimuli, including UV irradiation and treatment with anti-cancer drugs such as cisplatin and mitomycin C^8^^,^^9^. Because the nuclear localization of YB-1 is reversible, and YB-1 or its homologues associate with mRNAs translocating into the cytoplasm, YB-1 is considered to be a nucleo-cytoplasmic shuttling protein^21,22^. YB-1 is highly expressed in a variety of human cancers, and its nuclear localization is associated with tumor malignancy^7^^,^^9^^,^^23^. YB-1 activates the transcription of genes involved in cell proliferation or tumor progression, including the multi-drug resistance 1, cyclins, EGF receptor, and metalloproteinase 9 (reviewed by^9^). Therefore, the subcellular localization of YB-1 must be tightly regulated, and could serve as a potential therapeutic target in many human cancers.

We have identified the Y-box protein–associated acidic protein (YBAP1) in chicken DT40 cells as well as in Xenopus oocytes^24^^,^^25^. Chicken YBAP1 is equivalent to chicken 38 K protein and a homologue of human ASF/SF2-associated protein p32, C1QBP (complement component 1, q subcomponent binding protein, also called gC1qR), and hyaluronan-binding protein 1^26,27^^,^^28^. YBAP1/C1QBP has been reported to localize at the cell surface, or in the cytoplasm, the mitochondria, or the nucleus^27,29,30^. YBAP1 forms a protein-protein complex with YB-1 and thereby removes some YB-1 molecules from YB-1-mRNA complexes^24^. This mRNP remodeling relieves translational repression caused by YB-1. Interaction between YB-1 and YBAP1/C1QBP, or their orthologues, has also been reported in humans and other organisms^31–33^.

In this study, we found that YBAP1/C1QBP binds at least two regions in the C-terminal domain of YB-1, one of which overlaps with the NLS of YB-1. In cultured cells, co-overexpression of YBAP1 repressed the nuclear localization of a point mutant of YB-1. In addition, the YBAP1 binding fragment of YB-1 was imported by the nuclear receptor transportin 1 in a semi-intact cell system and YBAP1 competitively inhibited the interaction between YB-1 and transportin 1. In the cytoplasm, YBAP1 affects the accumulation of YB-1 to P-bodies and partially abrogated the mRNA stabilization by YB-1. Our results suggest that the interaction with YBAP1/C1QBP regulates the localization and cytoplasmic activities of YB-1.

## Results

### YBAP1/C1QBP binding domains in the YB-1 C-terminal tail domain

**Figure 1:**
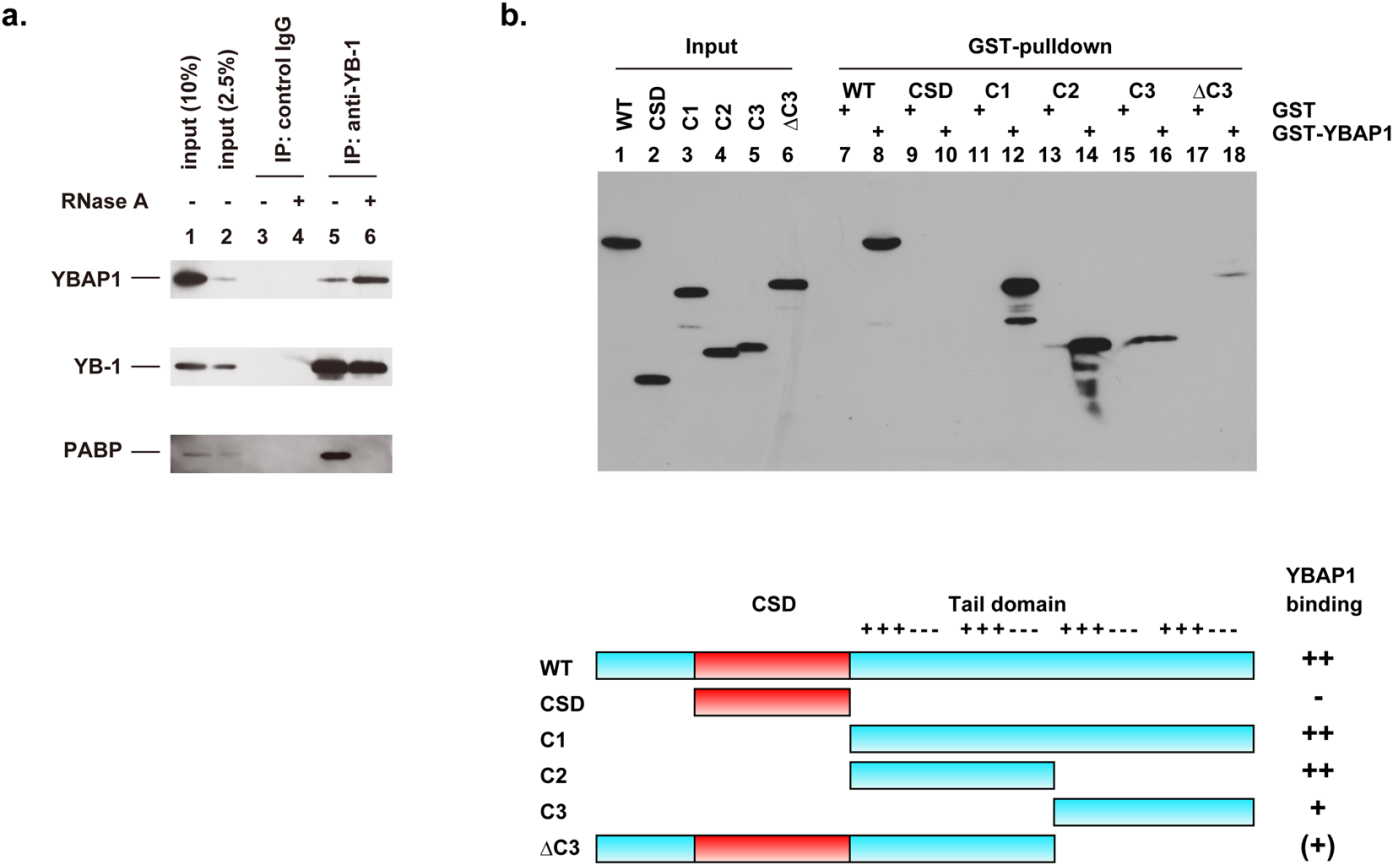
YBAP1 interacts with the C1 and C2 regions of the YB-1 tail domain. (**a**) HeLa cell lysates were treated with (lanes 4 and 6) or without (lanes 3 and 5) RNase A, and subjected to immunoprecipitation with control rabbit IgG (lanes 3 and 4) or rabbit anti-YB-1 antibodies (lanes 5 and 6). Immunoprecipitates and cell lysates (10% of input, lanes 1; 2.5% of input, lane 2) were analyzed by immunoblotting with antibodies against YB-1, YBAP1, and PABP. (**b**) FLAG-tagged YB-1 and its fragments were synthesized in wheat germ extracts and incubated with GST (lanes 7, 9, 11, 13, 15, and 17) or GST-YBAP1 (lanes 8, 10, 12, 14, 16, and 18). Protein complexes were bound to glutathione–Sepharose, and the eluted materials together with the input extracts (lanes 1–6) were analyzed by immunoblotting with anti-FLAG antibodies. Schematic diagrams of the YB-1 fragments are shown at the bottom. +++ and −−− indicate basic and acidic/aromatic amino acid clusters, respectively.

We previously identified YBAP1 as a co-immunoprecipitant with FLAG-tagged YB-1 expressed in YB-1^−/−^ cultured cells^24^. In this study, to determine whether endogenous YB-1 interacts with endogenous YBAP1 in HeLa S3 cells, we performed immunoprecipitation with anti-YB-1 antibody (Fig. 1a). YBAP1 was co-immunoprecipitated with YB-1 from the cell lysates. When the cell lysates were pretreated with RNase A, the amount of YBAP1 that co-precipitated with YB-1 increased, suggesting that YBAP1 interacts with YB-1 more efficiently when RNA is destroyed than when RNA is present. By contrast, PABP was co-precipitated with YB-1 only when RNA is preserved, consistent with the current model that YB-1 and PABP, as major constituents of mRNPs, interact with each other via mRNA^8^^,^^34^.

Our previous study showed that YBAP1 interacts with the C-terminal tail domain of YB-1^24^. To further characterize the YBAP1 binding domain(s) in the YB-1 tail domain, we expressed several deletion mutants of YB-1 in an *in vitro* translation system and subjected them to a glutathione-S-transferase (GST)-pulldown assay with GST-YBAP1 (Fig. 1b). Full-length YB-1 (wild-type, WT) and the tail domain (C1) associated with YBAP1, whereas the CSD did not, as we showed previously^24^. We found that the first two alternating A/B regions of acidic and basic clusters, which we here refer to as the C2 region, bound to YBAP1 very efficiently, whereas a deletion mutant (C3) consisting of the last two alternating A/B regions could also independently associate with YBAP1, albeit to a lesser extent. The basic amino acid clusters in the C2 and C3 regions are likely responsible for binding to YBAP1, which is acidic. Interestingly, a C-terminal deletion mutant (ΔC3) lacking the last two alternating A/B regions only weakly bound to YBAP1, although it contains the C2 region, suggesting that the N-terminal domain or CSD negatively regulates the binding of YBAP1 to the C2 region. Although the CSD has a well-packed structure, the N-terminal and the tail domains of YB-1 are believed to be intrinsically disordered, and might be stabilized by binding to ligands such as RNA or YBAP1. Nevertheless, structural characterization in solution revealed that the entire YB-1 molecule is relatively compact, suggesting an intramolecular interaction between the CSD and the N-terminal and the tail domains^35,36^. Consistent with this, the fibril formation of the CSD is prevented by the first half of the tail domain, which corresponds to the C2 region in this study^37^. We postulate that, the binding of YBAP1 to the C2 region is hampered by the N-terminal domain or the CSD in our ΔC3 mutant in a similar manner to inhibition of the CSD fibril formation. Further deletion analysis showed that two basic amino acid clusters were necessary for strong association with YBAP1 (C6, Supplementary Fig. S1). Taken together, these results indicate that there are at least two binding sites for YBAP1 in the YB-1 tail domain, and that the C2 region has the stronger binding activity.

### YBAP1 inhibits the nuclear localization of YB-1 mutants expressed in cells

**Figure 2:**
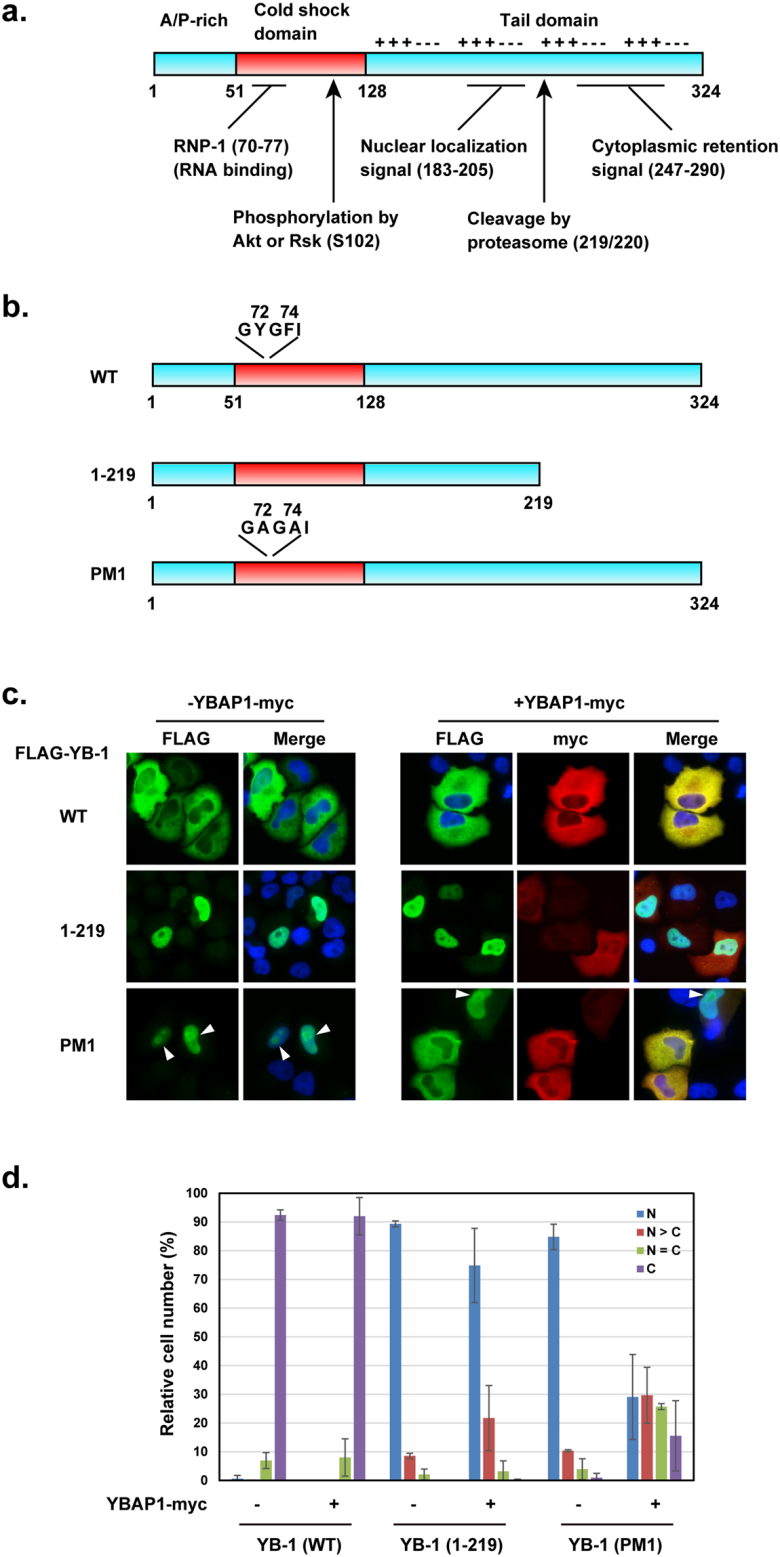
YBAP1 affects nuclear localization of YB-1. (**a**) Domain structure of YB-1 protein. (**b**) Schematic diagrams of wild-type, the 1–219 deletion mutant, and the point mutant PM1. The consensus amino acid sequences of RNP-1 in the CSD, which were mutated in PM1, are shown. (**c**,**d**) FLAG-YB-1 expression vectors pCMV-3xFLAG-YB-1 (WT, 1-219 and PM1) were transfected into HeLa cells with or without the YBAP1-myc expression vector pCI-neo-C1QBPmyc. Cells were immunostained with antibodies against FLAG (green) and c-myc (red) tags, and stained with DAPI. Nucleoli are indicated by white arrowheads (**c**). Localization of FLAG-YB-1 was examined for >100 cells and classified into four categories (N, signals restricted to the nuclei; N > C, nuclear signals stronger than cytoplasmic signals; N = C, equal intensity of cytoplasmic and nuclear signals; C, cytoplasmic signals stronger than nuclear signals). In cells co-transfected with YBAP1-myc expression plasmid, localization of FLAG-YB-1 in cells co-expressing YBAP1-myc was assessed. The quantitative results of three independent experiments are shown as relative cell number (%) in d. Bars, s.d.

Previous studies identified an NLS (aa 183–205, Fig. 2a) and a cytoplasmic retention signal (CRS, aa 247–290) in the YB-1 C-terminal tail domain, and showed that the YB-1 CRS functionally prevails over the NLS^14,38,39^. Based on our observation that YBAP1 binds to the C2 region containing this NLS, we sought to examine the effect of YBAP1 on the subcellular localization of YB-1. To do this, we expressed FLAG-tagged YB-1 mutants in HeLa S3 cells and examined the effect of co-expressed YBAP1 on their localization. We prepared expression vectors for wild-type YB-1, a truncated YB-1(1–219) that corresponds to the N-terminal product of proteasomal degradation^39^, and a point mutant, PM1 (Fig. 2b). PM1 has two amino acid substitutions (GYGFI → GAGAI) in the RNP-1 motif of the CSD that significantly weaken the RNA-binding activity of the Y-box protein, although the mutant retains translation inhibition activity^40^^,^^41^.

When expressed in HeLa S3 cells without co-expression of YBAP1, wild-type YB-1 was detected in the cytoplasm in most cells, but distributed in both nuclei and cytoplasm in less than 10% of cells (Fig. 2c left panels and 2d). These results likely reflect the nucleo-cytoplasmic shuttling of YB-1. When co-expressed with YBAP1-myc, the localization of wild-type YB-1 was not affected (Fig. 2c right panels and 2d). Without co-expressed YBAP1, YB-1(1–219) and PM1 mutants were detected exclusively in the nuclei in 89% and 85% of transfected cells, respectively, with PM1 accumulating in the nucleoli. In most cells, YB-1(1–219) remained in the nucleus when co-expressed with YBAP1. Interestingly, the localization of PM1 in cells differed significantly in the presence or absence of YBAP1: specifically, in cells co-expressing YBAP1, PM1 was detected in the cytoplasm in 70% of cells. Notably, PM1 distributed both to the nuclei and the cytoplasm in 25% of cells, and exhibited exclusively cytoplasmic localization in 15% of cells (Fig. 2d). These results demonstrate that overexpression of YBAP1 induces a shift in the localization of YB-1 to the cytoplasm.

### Transportin 1 mediates the nuclear import of the YB-1 C2 region

**Figure 3:**
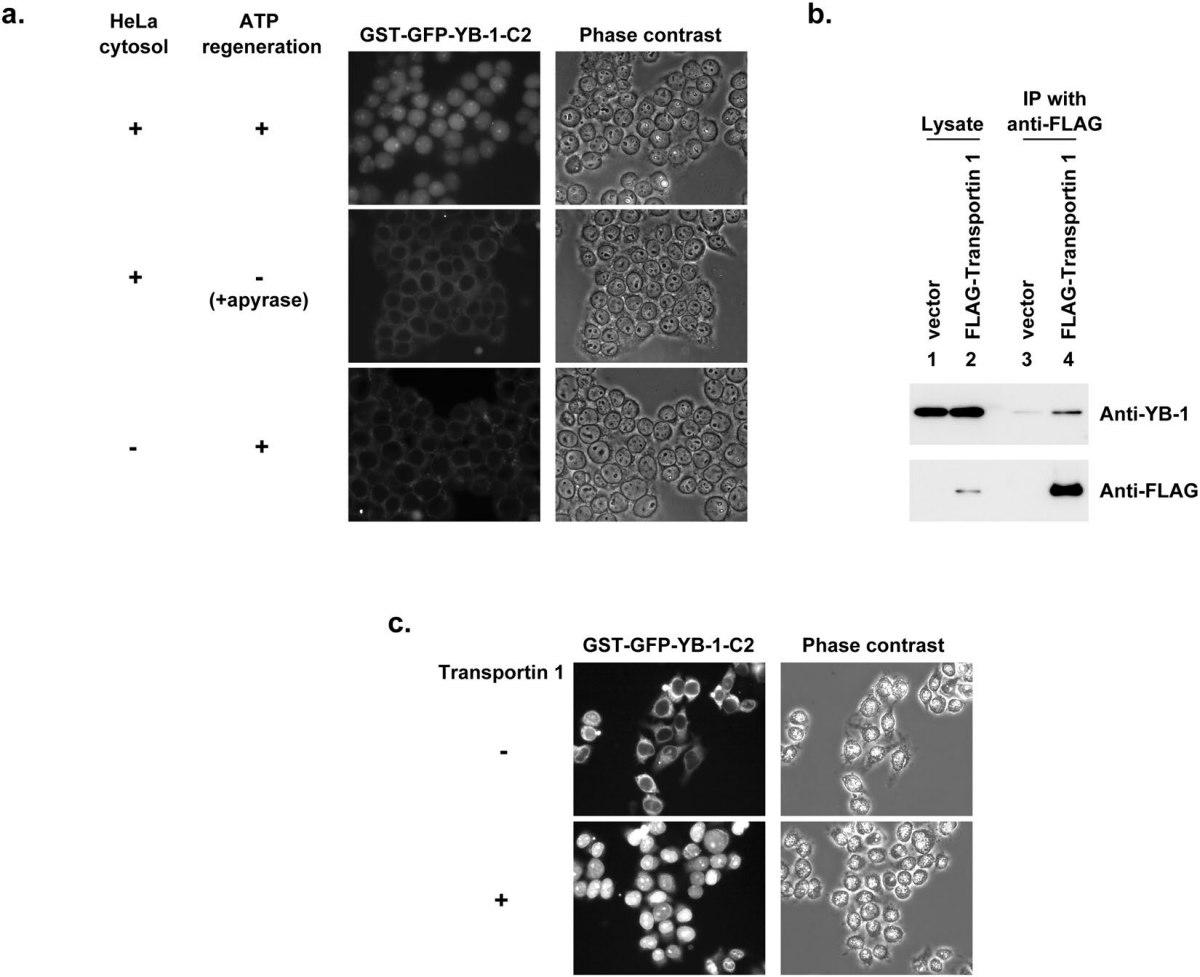
Transportin 1 is the nuclear import receptor for YB-1. (**a**) Nuclear localization of GST-GFP-YB-1-C2 was examined in a semi-intact cell system with HeLa cytosol. Digitonin-permeabilized HeLa cells were incubated with GST-GFP-YB-1-C2, HeLa cytosolic fraction, and an ATP regeneration system. Localization of GST-GFP-YB-1-C2 was examined by fluorescence microscopy (left panels). Phase-contrast images are also shown (right panels). In some experiments, ATP regeneration system was omitted, and apyrase was included instead (middle row). (**b**) FLAG-Transportin 1 expression vector, pCMV-3xFLAG-TRN1 (lanes 2 and 4), or empty vector (lanes 1 and 3) was transfected into HeLa cells. Two days later, cell lysates were subjected to immunoprecipitation with anti-FLAG antibodies. Immunoprecipitates and input lysates were analyzed by immunoblotting with antibodies against YB-1 and FLAG. (**c**) Permeabilized HeLa cells were incubated with 2 μM GST-GFP-YB-1-C2 with 4 μM Ran-GDP and an ATP regeneration system in the absence (top row) or presence (bottom row) of 0.5 μM transportin 1. Localization of GST-GFP-YB-1-C2 was examined by fluorescence microscopy (left panels). Phase-contrast images are also shown (right panels).

We next sought to identify the nuclear import receptor of YB-1. Because the C2 region contains the NLS, we first investigated whether the C2 region is imported into the nuclei in a semi-intact cell system. This system utilizes HeLa cytosol fraction as a source of nuclear import receptor(s), and an ATP regeneration system, both of which are required for transport of cargo into the nuclei of digitonin-permeabilized HeLa cells^5^. We prepared the recombinant protein GST-GFP-YB-1-C2, in which the C2 region is fused to GST, which facilitates affinity purification, and to GFP, which can be detected by fluorescence microscopy. When GST-GFP-YB-1-C2 was subjected to the semi-intact cell system, the protein was efficiently imported into the nuclei (Fig. 3a). The nuclear import of GST-GFP-YB-1-C2 was dependent on the cytosolic fraction and inhibited by energy depletion, as assessed by the addition of apyrase, which catalyzes the hydrolysis of ATP. These results indicate that the semi-intact cell system can recapitulate the nuclear import of the C2 region of YB-1.

The NLS in the C2 region is classified as a basic PY-NLS, an NLS consisting of a basic amino acid–rich region with a C-terminal proline–tyrosine sequence. This class of NLS interacts with the nuclear import receptor transportin 1/karyopherin β2^42–44^. In their previous work, Lee *et al*. demonstrated that GST-YB-1(149–203) containing the PY-NLS interacts with transportin 1, and that this interaction can be inhibited by excess Ran-GTP^43^. To test whether endogenous YB-1 interacts with transportin 1, we expressed FLAG-transportin 1 in HeLa cells, and subjected cell lysates to immunoprecipitation with anti-FLAG antibody. Western blotting of immunoprecipitates with anti-YB-1 antibody revealed that endogenous YB-1 co-immunoprecipitated with transportin 1 at a significantly higher level than in a control immunoprecipitation from cells transfected with an empty vector (Fig. 3b). Therefore, we next investigated whether transportin 1 mediates the nuclear import of the C2 region in a reconstituted semi-intact cell system consisting of purified proteins (Fig. 3c). When digitonin-permeabilized cells were incubated with GST-GFP-YB-1-C2 with Ran-GDP and an ATP regeneration system in the presence of recombinant transportin 1, GST-GFP-YB-1-C2 was imported into the nuclei in a transportin 1–dependent manner. These results indicate that transportin 1 mediates the import of the C2 region into the nuclei. During preparation of this manuscript, Mordovkina *et al*. reported that YB-1 nuclear import in permeabilized cells is inhibited by the M9M peptide, which sequesters transportin 1 and thus acts as an inhibitor of transportin 1–mediated nuclear import; in addition, they showed that alanine substitutions in the PY motif (aa 201 and 202) diminish the nuclear import^45^. Our results, together with their work, provide direct evidence for the role of transportin 1 in the nuclear import of YB-1.

### YBAP1 inhibits the nuclear import of the YB-1 C2 region by transportin 1

**Figure 4:**
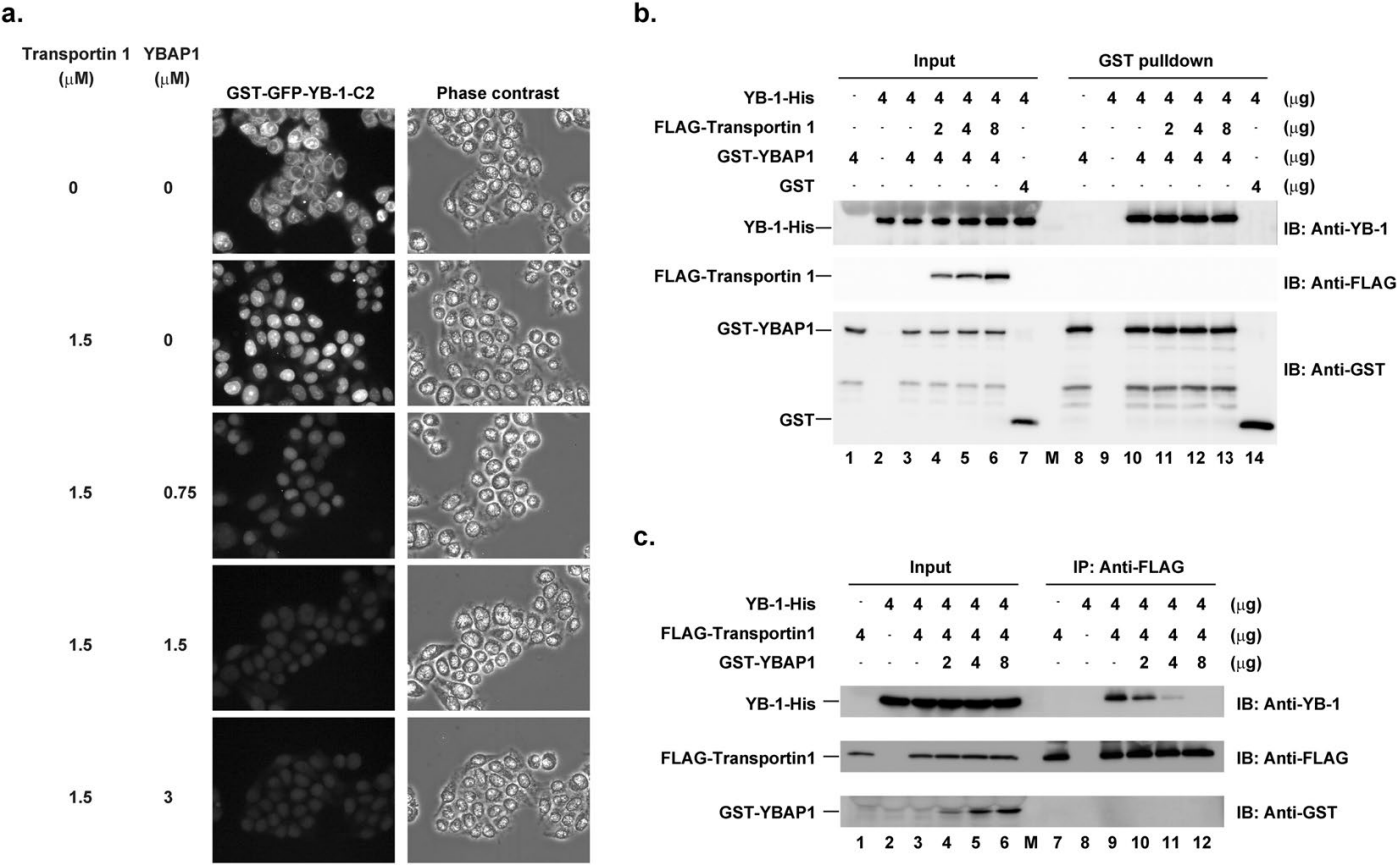
YBAP1 inhibits nuclear import of the YB-1 C2 region by transportin 1. (**a**) Permeabilized HeLa cells were incubated with 1.6 μM GST-GFP-YB-1-C2, 1.5 μM transportin 1, 4 μM Ran-GDP, and an ATP regeneration system in the presence of 0–3 μM YBAP1. Localization of GST-GFP-YB-1-C2 was examined by a fluorescence microscopy. (**b**) YB-1-His, GST-YBAP1 and GST were incubated with increasing amounts of FLAG-transportin 1. The mixture was subjected to GST-pulldown assays as described in Methods. The eluted proteins (lanes 8–14) and aliquots of the reaction mixture (input, lanes 1–7) were then analyzed by immunoblotting. (**c**) YB-1-His and FLAG-transportin 1 were incubated with increasing amounts of GST-YBAP1. The mixture was subjected to immunoprecipitation with anti-FLAG antibodies as described in Methods. The eluted proteins (lanes 7–12) and aliquots of the reaction mixture (input, lanes 1–6) were analyzed by immunoblotting.

Given that YBAP1 bound to the C2 region (Fig. 1) and affected the localization of YB-1 in cells (Fig. 2), we next examined whether YBAP1 would also affect the nuclear import of the YB-1-C2 region by transportin 1 in the semi-intact cell system. When digitonin-permeabilized cells were incubated with GST-GFP-YB-1-C2 in the presence of transportin 1, Ran-GDP, and an ATP regeneration system with increasing amounts of YBAP1, the nuclear import of GST-GFP-YB-1-C2 was inhibited in a dose-dependent manner (Fig. 4a). By the addition of a 2-fold molar excess of YBAP1 relative to transportin 1, the import of GST-GFP-YB-1-C2 was almost completely inhibited.

To elucidate the molecular mechanism of nuclear import inhibition by YBAP1, we examined the effect of YBAP1 on the interaction between YB-1 and transportin 1, and vice versa. When YB-1 was incubated with GST-YBAP1, but not with GST itself, YB-1 was co-precipitated with GST-YBAP1 by glutathione–Sepharose (Fig. 4b). The addition of increasing amounts of FLAG-transportin 1 to the mixture did not affect the amount of YB-1 co-precipitated with GST-YBAP1, indicating that transportin 1 did not inhibit the interaction between YB-1 and YBAP1. In a complementary set of experiments, when YB-1 was incubated with FLAG-transportin 1, YB-1 was co-precipitated with FLAG-transportin 1 by anti-FLAG antibody (Fig. 4c). The addition of increasing amounts of GST-YBAP1 resulted in a significant decrease in the amount of YB-1 co-precipitated with FLAG-transportin 1. Based on these results, we conclude that YBAP1 inhibits the interaction between YB-1 and transportin 1.

### Overexpression of YBAP1 affects the YB-1 cytoplasmic localization

**Figure 5:**
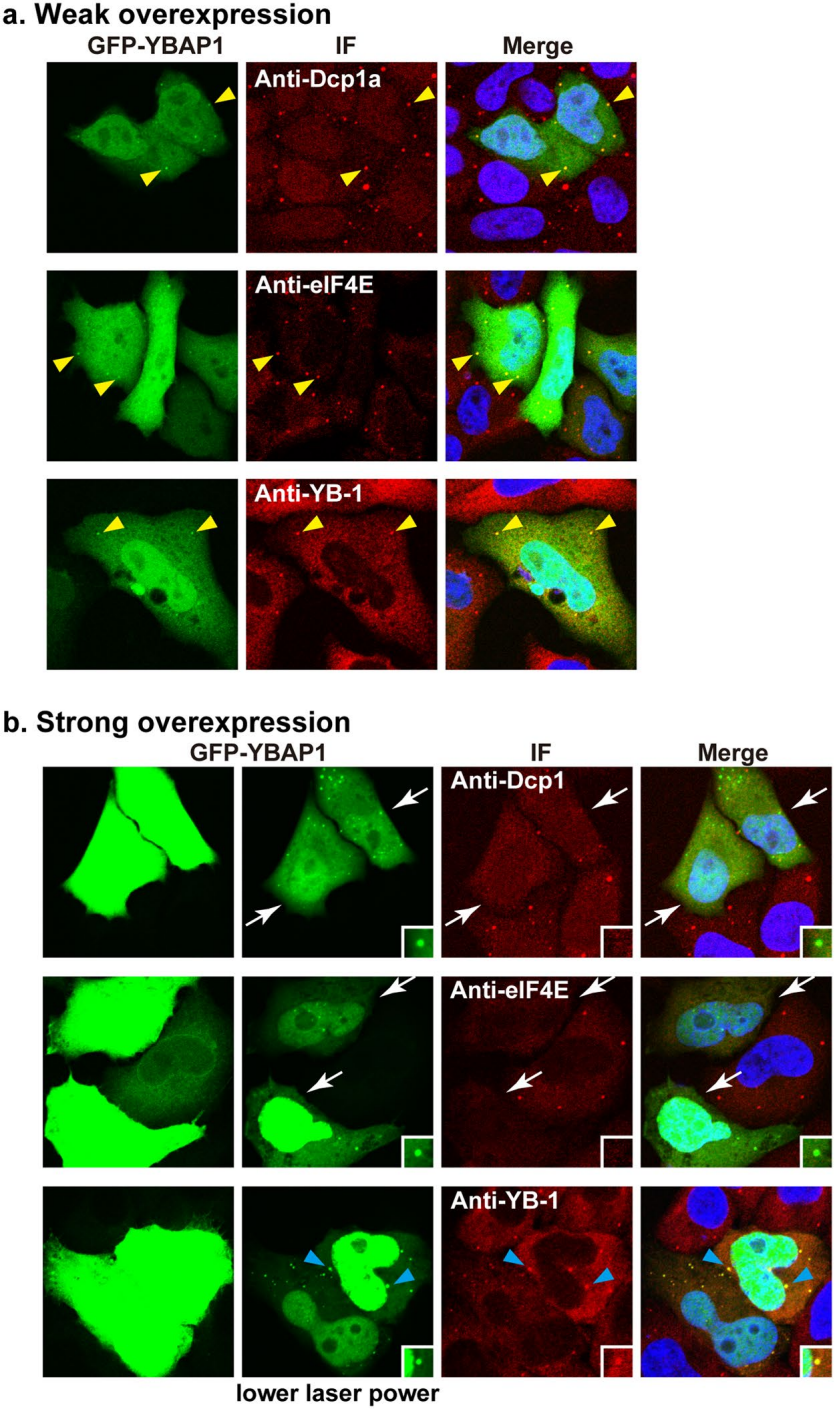
GFP-YBAP1 affects the YB-1 cytoplasmic localization. (**a**,**b**), HeLa cells were transfected with a GFP-YBAP1 expression vector, and two days later the cells were immunostained with antibodies against Dcp1a, eIF4E and YB-1 (red), counter-stained with TO-PRO-3 (blue) and examined under a laser confocal microscope. Cells with weakly overexpressed GFP-YBAP1 (green) are shown in a. and those with strongly overexpressed GFP-YBAP1 are shown in b. Photographs in the left columns in (**a**,**b**) were taken with the same laser power, while those in the second left column in (**b**) were with lower laser power. Insets show the cytoplasmic GFP-YBAP1 aggregates in (**b**). Yellow arrowheads in (**a**) indicate P-bodies. White arrows and blue arrowheads in (**b**) indicate the cells without P-bodies, and cytoplasmic aggregates where YB-1 and GFP-YBAP1 were co-localized.

As we have found that YBAP1 regulates nucleo-cytoplasmic distribution of YB-1, we then tested whether the YB-1 cytoplasmic localization is affected by GFP-YBAP1 expressed in HeLa cells. Immunofluorescence showed that YB-1 distributes diffusely in the cytoplasm and also localizes to P-bodies during interphase as reported previously (Fig. 5a,^19,20^). While weakly overexpressed GFP-YBAP1 distributed throughout the cells and was accumulated to P-bodies co-localizing not only with YB-1 but also with other known P-body components eIF4E and Dcp1a (Fig. 5a, yellow arrowheads), strongly overexpressed GFP-YBAP1 disrupted P-bodies (Fig. 5b, white arrows). Intriguingly, YB-1 co-localized with highly overexpressed GFP-YBAP1 in cytoplasmic aggregates that are irregularly-shaped and larger than P-bodies, which exemplifies the interaction between YB-1 and YBAP1 in cells (Fig. 5b, blue arrowheads). We are not sure whether these aggregates represent mRNA granules or protein aggregates. Given that GFP-YBAP1 was localized to P-bodies, we examined the localization of endogenous YBAP1. While endogenous YBAP1 was not detected in P-bodies at 37 °C, it accumulated to stress granules when cells were incubated at 44 °C for 30 min (Supplementary Fig. S2). These results suggest that YBAP1 can accumulate to mRNP granules, and that its overexpression affects cytoplasmic localization of YB-1. This prompted us to examine whether YBAP1 associates with mRNPs in HeLa cells. To this end, cell extracts either treated or not treated with RNase A were used for immunoprecipitation with antibodies against RAP55A, a cytoplasmic mRNP component^25^. YBAP1 was co-immunoprecipitated with RAP55A in an RNase-sensitive manner, while the interaction between RAP55A and another mRNP component RCK/p54/DDX6 was partially RNase-insensitive (Supplementary Fig. S3)^25^. These results, together with the localization analysis of YBAP1, suggest that YBAP1 associates weakly or transiently with mRNPs, although YBAP1 is not stably associated with mRNPs during sucrose gradient centrifugation^24^, and a small fraction of YBAP1 localizes to mRNP granules.

### YBAP1 partially abrogated the mRNA stabilization induced by YB-1

**Figure 6:**
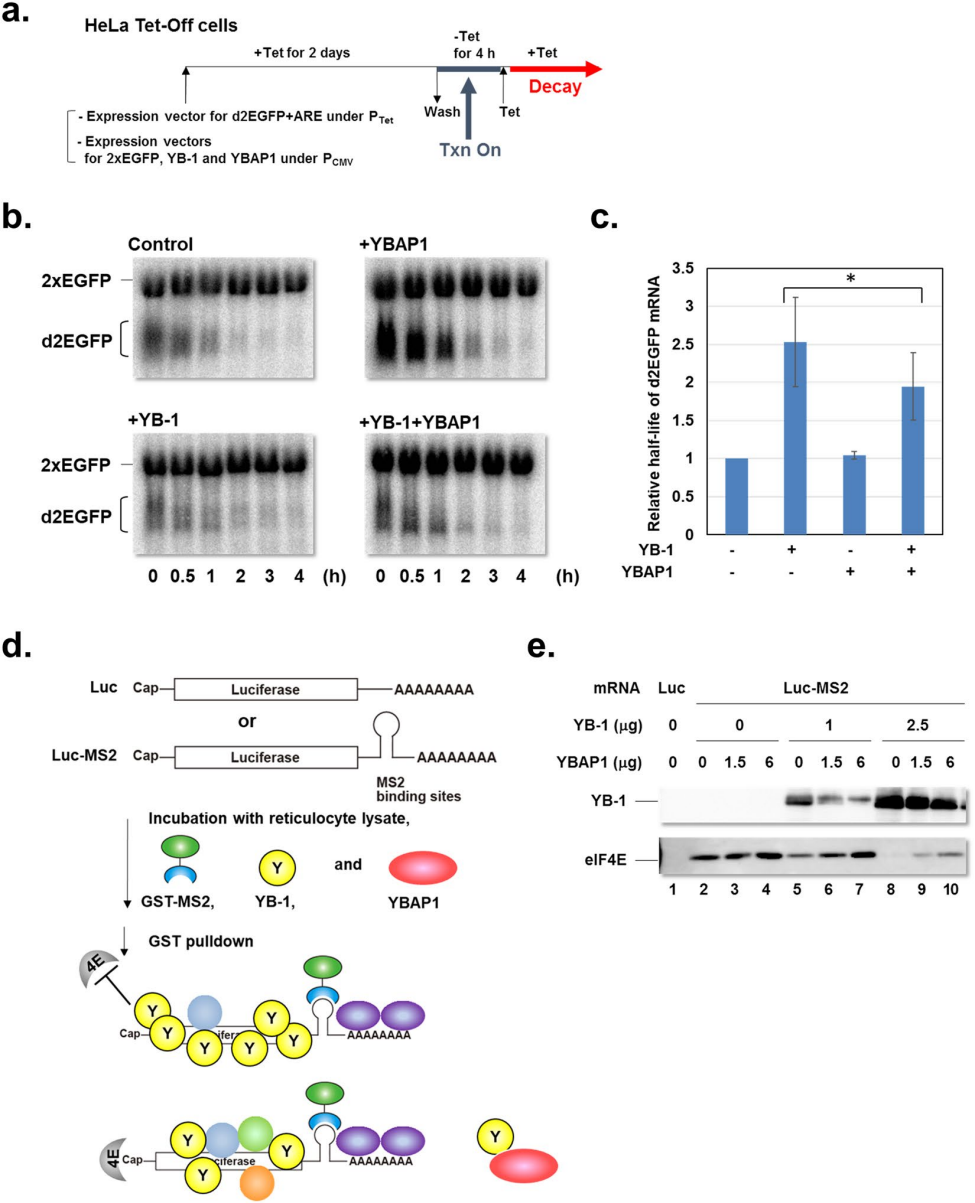
YBAP1 abrogates cytoplasmic functions of YB-1. (**a**) An experimental scheme to examine mRNA stability using a d2EGFP reporter mRNA in HeLa Tet-Off cells. (**b**) Cells were transfected with pTRE-d2EGFP-AU, pEGFP2C1, pCMV-YB-1-3xFLAG and pCI-neo-YBAP1. Transcription from the Tet-regulated promoter was induced for 4 h, and shut off by the addition of Tet. The cells were harvested at the indicated time points. d2EGFP mRNA and 2xEGFP mRNA as an internal control were detected by Northern blotting. (**c**) d2EGFP mRNA remaining at various time points after the Tet addition was quantified. Relative half-life of d2EGFP mRNA is shown. *P < 0.05. (**d**) An experimental scheme to examine the effect of YB-1 and YBAP1 on the mRNA binding of eIF4E. Purple ovals represent PABP. Light blue, green, and orange circles represent other mRNP proteins. (**e**) Luc mRNA (lane 1) or Luc-MS2 mRNA (lanes 2–10) was incubated with nuclease-treated RRL, YB-1-His, YBAP1-His, and GST-MS2. The mixture was subjected to GST-pulldown assays as described in Methods. The eluted proteins were then analyzed by immunoblotting.

The roles of YB-1 in the cytoplasm include translational activation or repression, and mRNA stabilization. In our previous work, we showed that YBAP1 abrogates the translation repression induced by YB-1^24^. Next we sought to demonstrate the function of YBAP1 in regulating the mRNA stabilization by YB-1. We then employed a reporter mRNA system in which a tetracyclin (Tet)-regulated expression vector for d2EGFP mRNA carrying an AU-rich element, which mediates rapid mRNA degradation, was transfected into HeLa Tet-Off cells (Fig. 6a). Transcription of the reporter was induced by the removal of Tet for 4 h, and then stopped by the addition of Tet. The mRNA stability was assessed by Northern blotting following the various time points after the Tet addition (Fig. 6b). The reporter d2EGFP mRNA was stabilized with overexpressed YB-1 (Fig. 6c). Overexpression of YBAP1 did not affect the reporter mRNA stability without YB-1, but partly attenuated the mRNA stabilization by overexpressed YB-1.

Packaging mRNA with the Y-box proteins stabilizes mRNA by precluding mRNA degradation enzymes and, in some cases, other mRNP components to gain access to mRNA^13,46^^,^^14^. YB-1 was reported to bind at or near the 5′ end of an mRNA, which hampers the association of the cap-binding protein eIF4E^14^. In order to test this in an mRNP context, naked firefly luciferase mRNA with MS2 binding sites in its 3′UTR (Luc-MS2 mRNA) was incubated with rabbit reticulocyte lysates (RRL) (Fig. 6d). mRNPs assembled in the RRL were incubated with GST-MS2 protein, and collected by glutathione Sepharose (Fig. 6e). Western blotting of the collected fractions revealed that eIF4E derived from the RRL was detected dependent on the MS2 binding sites, suggesting that the GST-pulldown experiments enabled us to detect the specific proteins, which bound to Luc-MS2 mRNA (Fig. 6e, lanes 1 and 2). When recombinant YB-1 was added to the mRNP assembly reactions, the amount of bound eIF4E was decreased (compare lanes 2, 5 and 8). With 2.5 μg of exogenous YB-1, eIF4E was barely detected, which likely reflects the inhibition of the mRNA binding of eIF4E via the binding of YB-1 to the proximity to the mRNA 5′ terminus. Under these conditions, a further addition of recombinant YBAP1 to the mRNP assembly reactions restored the amount of eIF4E in the collected fractions, concomitant with the decrease of YB-1 (e.g., compare lanes 5, 6 and 7). These results suggest that YBAP1 removed YB-1 from the 5′ proximity of an mRNA, which allowed eIF4E to gain access to the cap structure.

## Discussion

**Figure 7:**
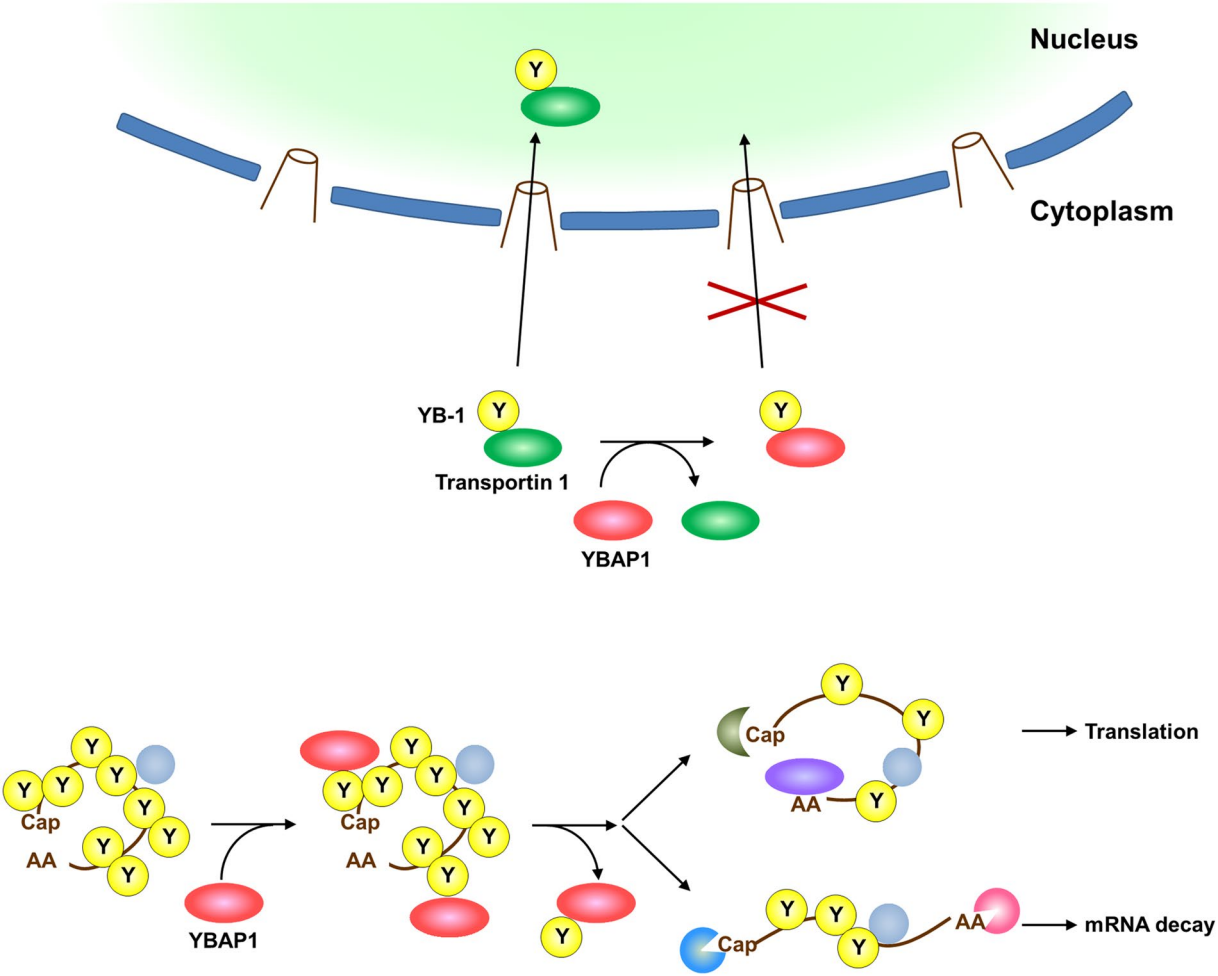
A schematic model for the role of YBAP1 in the regulation of YB-1 subcellular localization and mRNP dynamics.

The significance of nuclear YB-1 is demonstrated by the finding that levels of nuclear YB-1 is correlated with poor prognosis in a variety of human cancers (reviewed by^7^^,^^9^). These studies underscore the importance of the regulation of YB-1 nuclear translocation. In this study, we showed that the first two alternating A/B regions (C2 region) of YB-1 were imported into the nucleus by transportin 1, and that YBAP1/C1QBP bound to the same region (Figs 1 and 3). YBAP1 inhibited the interaction between YB-1 and transportin 1, and thus the nuclear localization of C2 region (Fig. 4). Previous work showed that YB-1 nuclear import is induced by phosphorylation of YB-1 Ser 102, proteolytic cleavage between aa 219 and 220 by the proteasome to remove the CRS, and stimuli such as UV irradiation and treatment with interferon gamma^39^^,^^47–49^, reviewed by^8^. In the pioneering work showing that UV irradiation induces YB-1 nuclear localization, the authors proposed the existence of a possible protein that anchors YB-1 to the cytoplasm^47^. The nuclear transport of YB-1 can indeed be regulated by other proteins or small molecules^50–52^. The splicing factor SRp30c, which interacts with YB-1, facilitates the YB-1 nuclear transport^50^. Ohashi *et al*. and Tanaka *et al*. identified several proteins as YB-1-NLS binding proteins; of these, HSP60 affects the nuclear transport of the first two alternating A/B regions of YB-1^51,53^. Our work provides a mechanistic explanation of how the YB-1-interacting protein can regulate the transportin 1–mediated nuclear import of YB-1 in the cell. Our results indicate that YBAP1 outcompetes transportin 1 for binding to YB-1 to repress the transportin 1–mediated nuclear import of YB-1 (Fig. 7). Thus, the YB-1 NLS seems to be masked by the interaction with YBAP1. Regulation of nuclear import of proteins by associated proteins is an established paradigm; one well-known example is NF- κB nuclear import, which is mediated by importin α/β and repressed by the complex formation with IκB^54^^,^^55^. Similarly, a negative regulator of nuclear import (NRNI), BRAP2, associates with p21 in the cytoplasm, which is required for the cytoplasmic localization of p21^56^^,^^57^. Nuclear import of Ci/Gli transcription factors is inhibited by Sufu (Suppressor of fused) in Hedgehog signaling^58^. Consistent with our data, it has been recently reported that knockdown of YBAP1/C1QBP is accompanied with the increase of nuclear translocation of YB-1 in a renal cell carcinoma cell line^59^.

We found that YBAP1 inhibited the nuclear localization of the YB-1 PM1 mutant, but not wild-type YB-1 (Fig. 2). PM1 harbors point mutations in the RNP-1 motif of CSD, significantly impairing the RNA-binding activity of this domain^14,40^. This defect most likely induces nuclear accumulation of the PM1 mutant of the Y-box proteins, because it cannot be incorporated into mRNP fractions in the cytoplasm^41^ and, once transported into the nucleus, it cannot stably associate with (pre-)mRNAs that would otherwise carry it back to the cytoplasm. By contrast, wild-type YB-1 is mostly detected in the cytoplasm, but is able to shuttle between the cytoplasm and the nucleus. We speculate that the shuttling of wild-type YB-1 to the nucleus is inhibited by YBAP1, once wild-type YB-1 associated with mRNAs is transported to the cytoplasm.

In addition to the inhibition of the YB-1 nuclear import, YBAP1 affected the YB-1 localization in the cytoplasm (Fig. 5). The weakly expressed YBAP1 localized to P-bodies, whereas the highly overexpressed YBAP1 disrupted the P-bodies, which is a shared phenotype of several P-body components that consist cytoplasmic mRNPs^60^, raising the possibility that YBAP1 serves as an mRNP component. The RNA-dependent association of YBAP1 with an mRNP component RAP55A supports this notion. However, our previous data have shown that YBAP1 is fractionated not in mRNP fractions but at the top fractions after sucrose gradient centrifugation^24^. Based on these results, we propose that YBAP1 is weakly or transiently associated with mRNPs possibly via YB-1. Interaction with YBAP1 affects the amount of active YB-1 either in the cytoplasm or the nucleus, and also might affect various functions of YB-1 in gene expression (Fig. 7). Indeed, we showed that YBAP1 partly attenuated the mRNA stabilization by overexpressed YB-1 in HeLa cells, and that YBAP1 restored the amount of eIF4E, concomitant with the decrease of the YB-1 amount, in mRNP fractions (Fig. 6). Perhaps this might reflect the removal of YB-1 from mRNA by YBAP1 as shown by gel retardation assay and by sucrose gradient centrifugation analysis^24^. The data shown here provide further evidence that supports our previous proposal that YBAP1 or other proteins associating with the Y-box proteins may act as an mRNP remodeling factor^24,61^. The changes in the composition of mRNPs provide important cues to regulate the fate of mRNAs, including their stability, splicing and translation activities^34,62–64^. Our data in this study showed that YBAP1/C1QBP regulates the nucleo-cytoplasmic distribution of YB-1 and its cytoplasmic functions.

## Methods

### Plasmids

C-terminally FLAG-tagged YB-1 cDNAs (wild-type (WT), CSD, C1, C2, C3 and ΔC3) for *in vitro* translation were amplified by PCR and inserted into pTNT (Promega). To generate FLAG-YB-1 expression vectors (WT and 1–219) for expression in mammalian cells, the corresponding cDNA sequences were amplified by PCR. To obtain YB-1 (PM1) cDNA, two rounds of PCR amplification were performed. In the first PCRs, two primer sets, primers A (5′-CGCGGAAGATCTAAGCTTATGAGCAGCGAGGCCGAGACCC-3′) and B (5′-TGTTGATGGCACCAGCTCCGTTCCTTACATTGAACC-3′), and primers C (5′-GAACGGAGCTGGTGCCATCAACAGGAATGACACC-3′) and D (5′-GGACGCGGATCCTTACTCAGCCCCGCCCTGCTCAGCCTCGGGAG-3′), were used to amplify YB-1 cDNA fragments. The products of the primary reactions were mixed and used for secondary PCR amplification with primers A and D. The WT, 1–219, and PM1 cDNAs were then cloned into p3xFLAG-CMV-10 (Sigma) to obtain pCMV-3xFLAG-YB-1 (WT, 1–219, and PM1, respectively). To generate pCI-neo-myc, two oligonucleotides (5′-GGCCTTGAACAAAAACTTATTTCTGAAGAAGATCTGTAGGC-3′ and 5′-GGCCGCCTACAGATCTTCTTCAGAAATAAGTTTTTGTTCAA-3′) were phosphorylated at their 5′ ends, annealed, and inserted into *Not*I-digested pCI-neo (Promega). YBAP1 cDNA was amplified by RT-PCR from total RNA from HeLa cells, and then cloned into pCI-neo-myc to yield pCI-neo-C1QBPmyc. To generate pGST-GFP-YB-1-C2, an expression plasmid for GST-GFP-YB-1-C2, the YB-1 C2 region (aa 129–229) was amplified by PCR and inserted into pGEX-6P-2/hGFP^65^. To generate the FLAG-transportin 1 expression plasmid, pCMV-3xFLAG-TRN1, the coding region of transportin 1 cDNA was inserted into p3xFLAG-CMV-10. To generate the YBAP1 and GFP-YBAP1 expression plasmids, pCI-neo-YBAP1 and pEGFP-YBAP1, the YBAP1 cDNA was cloned into pCI-neo and pEGFP-C1 (Clontech), respectively. To generate pTRE-d2EGFP-AU, oligonucleotides (5′-AATTCATTATTTATTATTTATTTATTATTTATTTATTTAAT-3′ and 5′-CTAGATTAAATAAATAAATAATAAATAAATAATAAATAATG-3′) were annealed, phosphorylated and inserted into the *Eco*RI-*Xba*I sites of pTRE-d2EGFP (Clontech). To generate a 2xEGFP expression plasmid, pEGFP2C1, the EGFP coding sequence was inserted into the *Bsp*EI site of pEGFP-C1. YB-1-FLAG and RAP55A-FLAG expression plasmids in p3xFLAG-CMV-14, pCMV-YB-1-3xFLAG and pCMV-hRAP55A-3xFLAG, were described previously^24^^,^^66^.

### Antibodies

Rabbit polyclonal antibodies against the N-terminal peptides of YB-1 were used^67^. Antibodies against Dcp1a and RCK were described previously^25^^,^^66^. Antibodies against YBAP1 described previously^68^ and those purchased from Affinity Bioreagents were used. Antibodies against PABP (Novus), eIF4E (Santa Cruz), FLAG (mouse monoclonal clone M2 and rabbit polyclonal antibodies, Sigma) and GST (GE Healthcare) were purchased from the indicated suppliers.

### Cell culture and immunocytochemistry

HeLa S3 cells were grown in Dulbecco’s modified Eagle’s medium (DMEM) supplemented with 10% fetal bovine serum (FBS) at 37 °C under an atmosphere containing 5% CO_2_. HeLa Tet-Off cells (Clontech) were grown in DMEM supplemented with Tet system approved FBS and 100 μg/ml G418. For immunostaining of protein antigens, cells were grown on glass coverslips. Cells were transfected with plasmid DNA using Lipofectamine 2000 (Life Technologies) as described previously^25^^,^^69^. After 24 h, the cells were washed with cold phosphate-buffered saline (PBS) and fixed for 20 min at room temperature with 4% paraformaldehyde in PBS. The fixed cells were washed with 0.3% Triton X-100 in PBS, and then blocked with 1% bovine serum albumin in PBS. After blocking, immunocytochemistry was performed as described^25^^,^^70^. The cells were examined under a laser confocal microscope equipped with a 63× objective lens (NA 1.40) (LSM510, Zeiss), or a fluorescence microscope (IX81, Olympus). Localization of FLAG-YB-1 was examined in at least 100 cells.

### Immunoprecipitation

To detect the interaction between endogenous YB-1 and YBAP1, HeLa cells were lysed by addition of buffer A (50 mM Tris-HCl [pH 7.5], 150 mM NaCl, 5 mM MgCl_2_, 0.5% Nonidet P-40, and Complete protease inhibitor cocktail [EDTA-free, Roche]) and passed three times through a 27-gauge needle. The cell lysates were incubated with the IgG fraction of polyclonal anti-YB-1 antibodies or control rabbit IgG bound to protein G–Sepharose (GE) for 1 h at 4 °C. The Sepharose beads was extensively washed with buffer B (50 mM Tris-HCl [pH 7.5], 150 mM NaCl, 40 mM EDTA, 0.5% Nonidet P-40), and the protein complexes were eluted with 700 μg/ml antigen peptide. The proteins were analyzed by immunoblotting.

To detect the interaction between YB-1 and transportin 1, HeLa cells were transfected with pCMV-3xFLAG-TRN1 or empty vector. Two days later, cells were harvested and lysed by the addition of buffer C (10 mM HEPES [pH 7.5], 50 mM β-glycerophosphate, 1% Triton X-100, 10% glycerol, 2 mM EDTA) containing 0.5% SDS and Complete protease inhibitor cocktail, and sonicated. The cell lysates were incubated with 20 μl of anti-FLAG M2 affinity gel (Sigma) for 2 h at 4 °C. The affinity gel was extensively washed with buffer C, and the protein complexes were eluted with 200 μg/ml 3xFLAG peptide (Sigma).

To examine the effect of YBAP1 on the YB-1–transportin 1 interaction, YB-1-His, GST-YBAP1, and FLAG-transportin 1 were prepared as described previously^24,71^. These recombinant proteins were incubated in 200 μl of buffer D (buffer A containing 1% bovine serum albumin) on ice for 20 min, followed by incubation with 20 μl of anti-FLAG M2 affinity gel for 1 h at 4 °C. The affinity gel was extensively washed with buffer D, and complexes containing FLAG-transportin 1 were eluted as above.

### GST pulldown

To determine the YBAP1 binding domain(s) in YB-1, *in vitro* translation was performed in a 50 μl reaction mixture containing TNT wheat germ extracts (Promega) and 1 μg of template plasmid DNA for 2 h at 30 °C. An aliquot of the reaction was incubated with 4 μg of GST or GST-YBAP1 in 450 μl of buffer E (50 mM Tris-HCl [pH 7.5], 150 mM NaCl, 20 mM EDTA, 0.5% Nonidet P-40) for 15 min at 30 °C, and then for 20 min on ice. The reaction was mixed with 20 μl of glutathione–Sepharose (GE) for 1 h at 4 °C. The Sepharose beads were extensively washed with buffer E and boiled in SDS gel sample buffer.

To examine the effect of transportin 1 on the YB-1–YBAP1 interaction, YB-1-His, GST-YBAP1, GST, and FLAG-transportin 1 were incubated as described in the previous section. The reaction was mixed with 20 μl of glutathione–Sepharose for 1 h at 4 °C. The Sepharose beads were extensively washed with buffer D, and complexes containing GST-YBAP1 or GST were eluted with 20 mM glutathione in 50 mM Tris-HCl (pH 8.0) for 5 min at room temperature.

To examine the effect of YB-1 and YBAP1 on the mRNA binding of eIF4E, capped Luc and Luc-MS2 mRNAs with A_60_ tails and YBAP-His protein were prepared as described previously^24^^,^^25^. GST-MS2 was kindly provided by Dr. Rei Yoshimoto^72^. Luc-MS2 mRNA was incubated in a 25 μl reaction mixture containing nuclease-treated RRL (5 μl, Promega), YB-1-His, YBAP1-His, and GST-MS2 for 10 min at 30 °C. The reaction was mixed with 30 μl of glutathione–Sepharose for 1 h at 4 °C. The Sepharose beads were extensively washed with buffer F (10 mM Hepes pH 7.5, 100 mM KCl) and boiled in SDS gel sample buffer.

### Semi-intact cell system

To obtain recombinant GST-GFP-YB-1-C2 protein, pGST-GFP-YB-1-C2 was transformed into *E*. *coli* strain BL21(DE3). GST-GFP-YB-1-C2 overexpressed in bacterial cells was purified through glutathione–Sepharose (GE Healthcare) as described previously^73^, and dialyzed against 20 mM HEPES (pH 7.3), 110 mM potassium acetate, 2 mM magnesium acetate, 5 mM sodium acetate, 0.5 mM EGTA, 1 mM dithiothreitol, and 0.5 mM phenylmethyl sulfonyl fluoride. Ran-GDP and HeLa cytosol fractions were prepared as described previously^5^^,^^74^. *In vitro* transport assays were performed essentially as previously described^5^. After protein mixtures containing GST-GFP-YB1-C2 proteins, as indicated in figure legends, were incubated with the permeabilized HeLa cells for 20 min at 30 °C, the cells were washed and fixed with 3.7% formaldehyde in transport buffer^5^. Nuclear fluorescent proteins were detected by fluorescence microscopy (Olympus BX51), and images were captured using an ORCA-ER camera (Hamamatsu) controlled by the MetaVue software (Universal Imaging).

### mRNA stability assay

HeLa Tet-Off cells were transfected with pTRE-d2EGFP-AU, pEGFP2C1, pCMV-YB-1-3xFLAG and pCI-neo-YBAP1. The cells were maintained for 2 days in the presence of 40 ng/ml Tet. The cells were then cultured for 4 h without Tet to induce transcription from the Tet-regulated promoter, and harvested at 0.5, 1, 2, and 4 h after transcription shut-off by the addition of Tet at a final concentration of 1 μg/ml. Total RNA was isolated with TRI reagent (Sigma), separated by electrophoresis in an agarose gel containing formaldehyde, and transferred to a positively charged Nylon membrane. d2EGFP mRNA and 2xEGFP mRNA as an internal control were detected by Northern blotting using the [^32^P]-labeled EGFP open reading frame as a probe. Signals were detected by an Image Analyzer BAS5000 (Fuji Film) and the quantification was performed with MultiGauge software.

### Data Availability

The data generated during the current study are available from the corresponding author on reasonable request.

## Electronic supplementary material

Supplemental information
