# Supplementary material for: Y-box protein-associated acidic protein (YBAP1/C1QBP) affects the localization and cytoplasmic functions of YB-1
Source: Sci Rep. 2018 Apr 18;8:6198. doi: 10.1038/s41598-018-24401-3 (PMC5906478; doi:10.1038/s41598-018-24401-3)
Supplement: Supplementary file 1 — Supplemental information [file 41598_2018_24401_MOESM1_ESM.pdf]

Supplementary information

Y-box protein-associated acidic protein (YBAP1/C1QBP) affects the localization and cytoplasmic functions of YB-1

Ken Matsumoto, Shingo Kose, Iku Kuwahara, Mami Yoshimura, Naoko Imamoto, and Minoru Yoshida

Figure S1. Deletion analysis of the YB-1 C-terminal domain required for association with YBAP1.

YB-1 cDNAs (C2, C4–C8) were amplified by PCR and inserted into pMal-c2X. Lysates from *E. coli* expressing maltose-binding protein (MBP)-fused YB-1 fragments expressed from these plasmids were subjected to GST-pulldown assays.

Figure S2. YBAP1 localizes to stress granules.

HeLa cells not treated (top row) or treated (bottom row) at 44°C for 30 min were immunostained with antibodies against YBAP1 (green) and eIF4E (red), counter-stained with TO-PRO-3 (blue) and examined under a laser confocal microscope.

Figure S3. RNA-dependent interaction of YBAP1 with RAP55A.

HeLa cells were transfected with pCMV-hRAP55A-3xFLAG or empty vector. The cell lysates were treated with (lanes 4 and 6) or without (lanes 3 and 5) RNase A, and subjected to immunoprecipitation with anti-FLAG M2 affinity gel. The cell lysates (lanes 1 and 2) and immunoprecipitates were analyzed by immunoblotting.

Figure S4-7. Uncropped images for Western blot and Northern blot experiments presented in Figures 1, 3, 4, 6, S1 and S3.

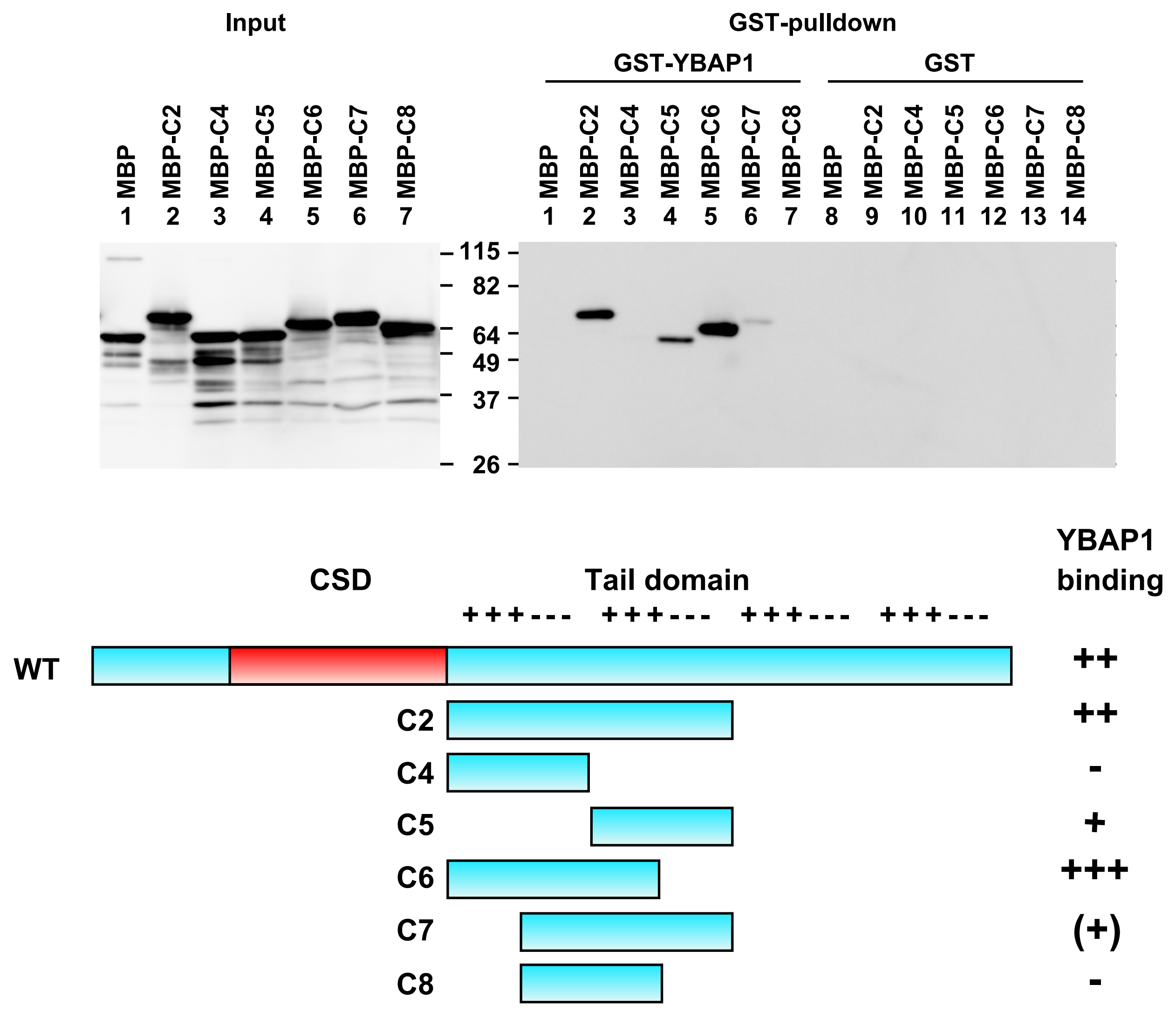
Input
GST-pulldown
GST-YBAP1
GST
MBP
MBP-C2
MBP-C4
MBP-C5
MBP-C6
MBP-C7
MBP-C8
1 2 3 4 5 6 7
1 2 3 4 5 6 7 8 9 10 11 12 13 14
115
82
64
49
37
26
CSD
Tail domain
+++--- +++--- +++--- +++---
YBAP1 binding
WT
C2
C4
C5
C6
C7
C8
++
++
-
+
+++
(+)
-

Figure S1

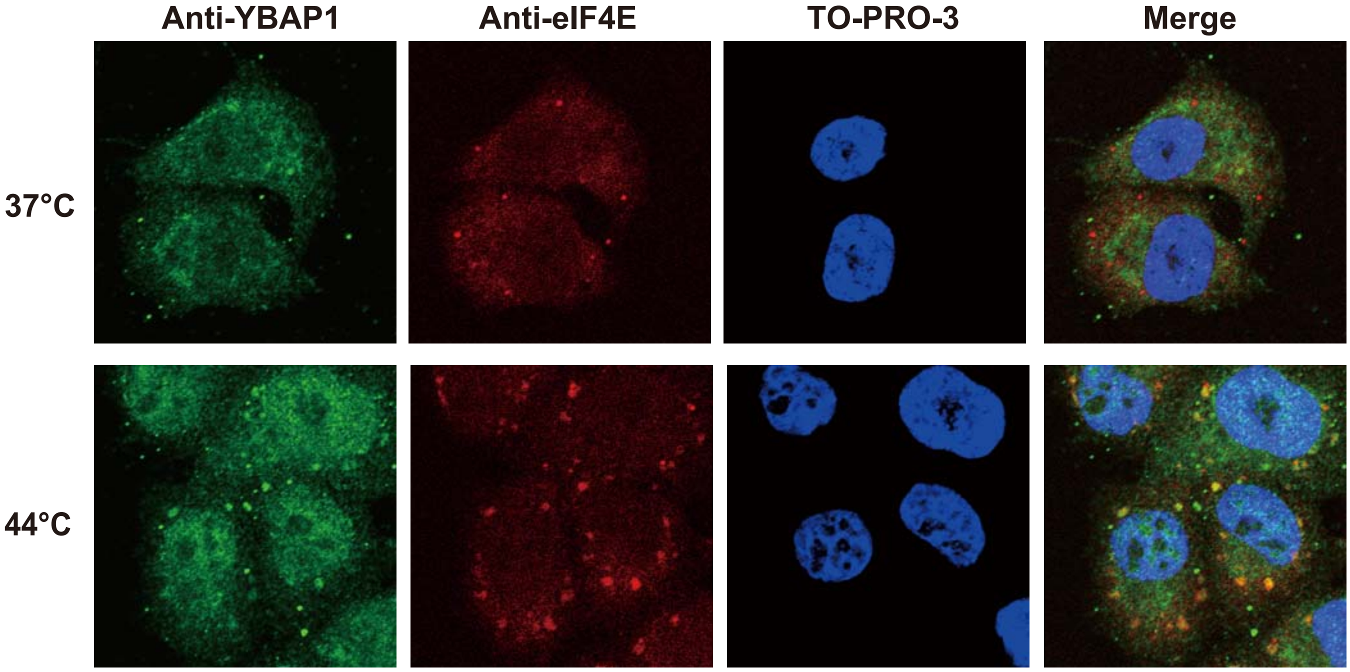

Figure S2

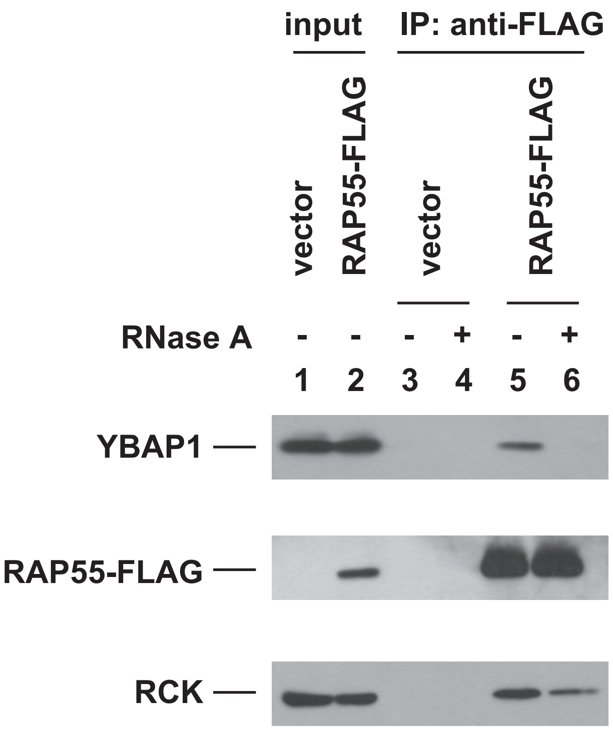
input
IP: anti-FLAG
vector
RAP55-FLAG
vector
RAP55-FLAG
RNase A
- - - + - +
1 2 3 4 5 6
YBAP1
RAP55-FLAG
RCK

Figure S3

Fig. 1a

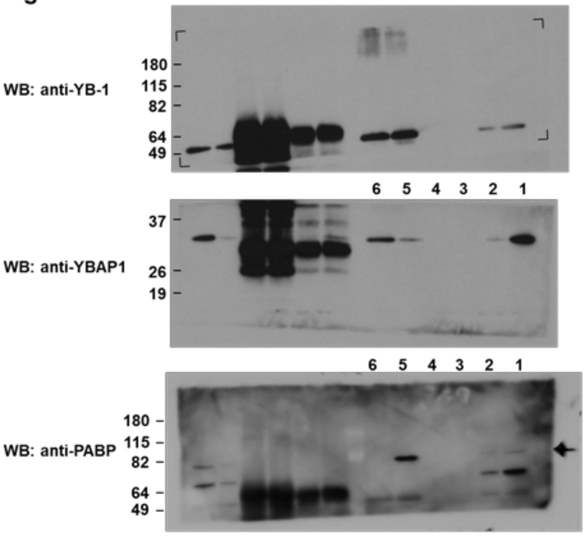

Fig. 1b

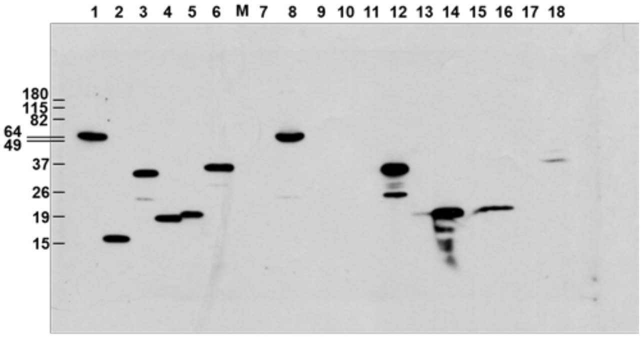

Fig. 3b

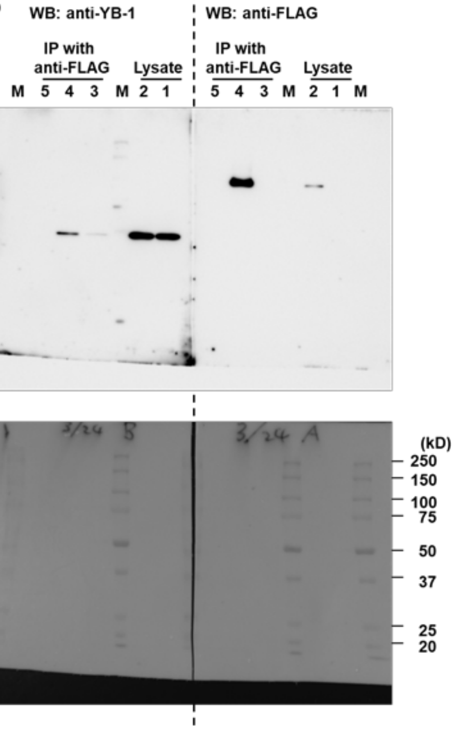

Figure S4

Fig. 4b

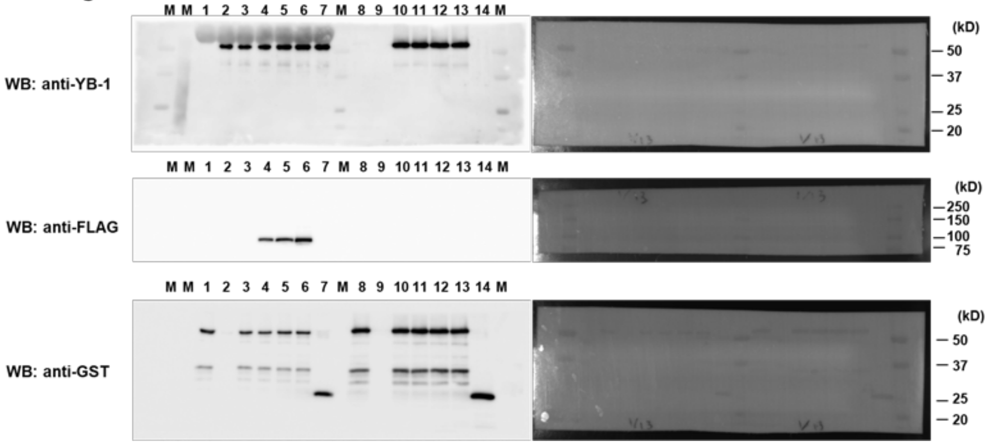

Fig. 4c

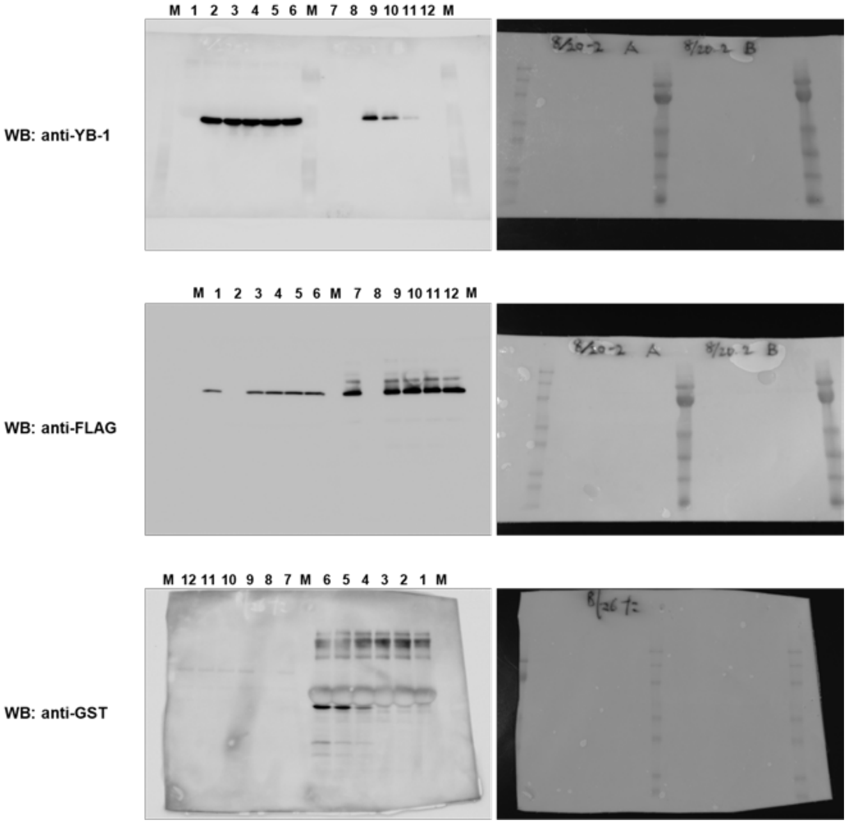

Figure S5

Fig. 6b

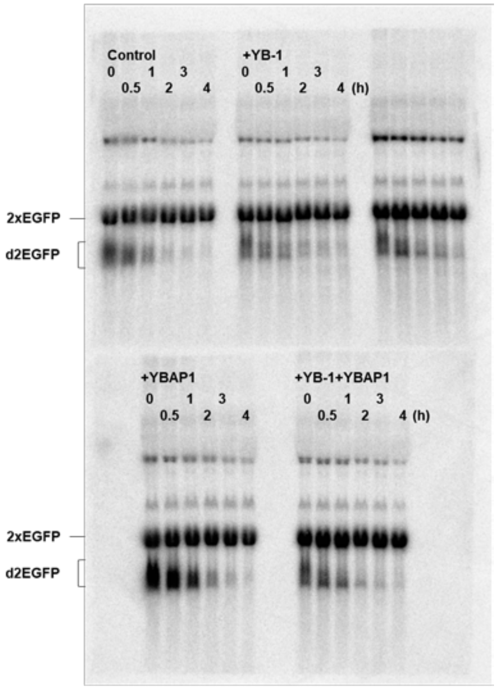

Fig. 6e

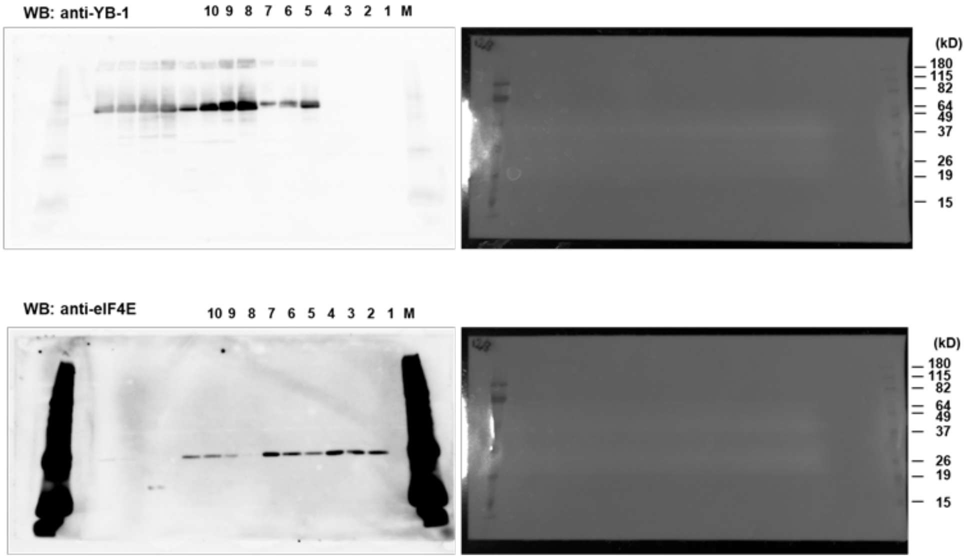

Figure S6

Fig. S1

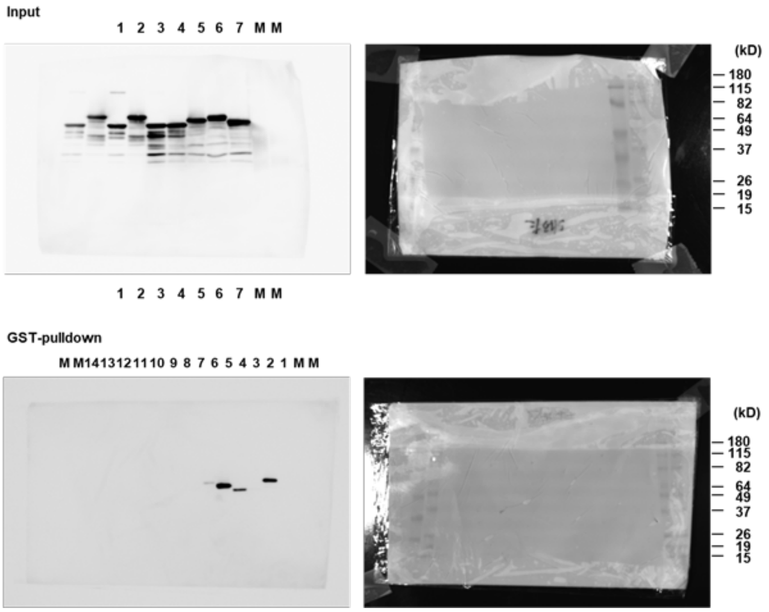

Fig. S3

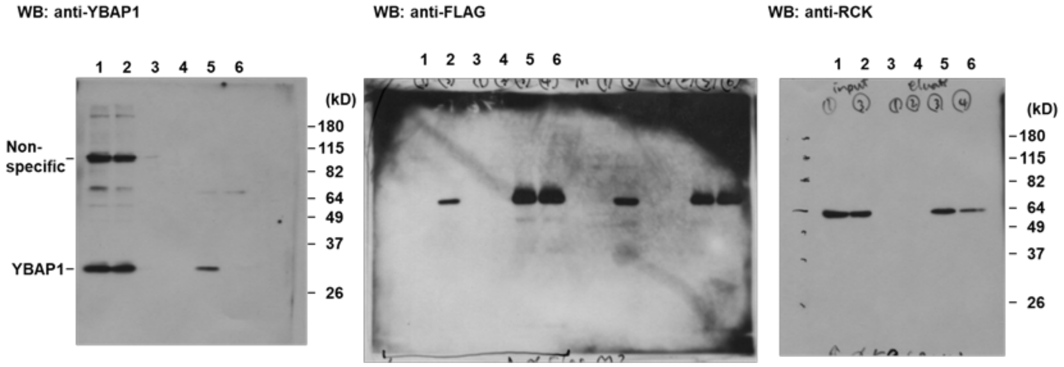

Figure S7
